# A New Real-time Method for Detecting the Effect of Fentanyl Using the Preoperative Pressure Pain Threshold and Narcotrend Index: A Randomized Study in Female Surgery Patients

**DOI:** 10.1097/MD.0000000000000316

**Published:** 2015-01-09

**Authors:** Guangyou Duan, Shanna Guo, Huiming Zhan, Dongmei Qi, Yuhao Zhang, Xianwei Zhang

**Affiliations:** From the Department of Anesthesiology, Tongji Hospital, Tongji Medical College, Huazhong University of Science and Technology, Wuhan, China.

## Abstract

Individual variability in the effects of opioid analgesics such as fentanyl remains a major challenge for tailored pharmacological treatment including postoperative analgesia. This study aimed to establish a new real-time method for detecting the effects of fentanyl and their individual differences in the preoperative period, using the pressure pain threshold (PPT) and Narcotrend index (NTI) test.

Eighty women undergoing elective surgery under general anesthesia were enrolled in this randomized, double-blinded, placebo-controlled study to receive either intravenous fentanyl (Group F) or saline (Group S). Before (T1) and 5 (T2) and 10 min (T3) after intravenous injection, the PPT, NTI, respiratory rate, heart rate, blood pressure, and pulse oxygen saturation were measured. The initial time at which the Narcotrend index showed a decline was also recorded.

In total, 40 patients in Group S and 38 patients in Group F were included in the final analysis. At 5 min and 10 min after intravenous fentanyl administration, the analgesic effect was determined by measuring the PPT, which was significantly increased (*P* < 0.001), and the sedative effect was detected using the NTI, which was significantly decreased (*P* < 0.001). The distribution of percentage changes of the PPT and NTI showed individual differences. At T2 and T3, the absolute changes in NTI and PPT were positively correlated (*r* = 0.444 at T2, *P* = 0.005; *r* = 0.332 at T3, *P* = 0.042).

Through the PPT and NTI, it was feasible to easily detect the effects of fentanyl and their individual differences in real time before induction of anesthesia in the operation room. This method could potentially be applied to preoperatively determine patients’ sensitivity to fentanyl.

## INTRODUCTION

Opioid analgesics are the most commonly used drugs for relief of postoperative pain in surgery patients. However, individual variability in analgesic efficacy is a major challenge in tailored pharmacological treatment.^1,2^ Fentanyl has a strong analgesic effect and is thus a widely used intravenous opioid analgesic for postoperative pain control.^3,4^ Implementation of appropriate preoperative methods for detecting the effects of opioid analgesics and their individual differences in advance would help to identify patients who are more or less sensitive to opioid analgesics such as fentanyl, thus facilitating the choice of more effective personalized pain treatment plans.^5,6^ However, at present, no feasible methods for real-time detection of the clinical effects of opioid analgesics including fentanyl exist; therefore, timely administration of personalized dosing of fentanyl remains unclear.

By measuring experimental pain, some studies have detected the effect of oral opioids such as morphine in patients with chronic pain,^7,8^ and similarly, a few studies have tested intravenous opioids such as pentazocine, morphine, and remifentanil, but not fentanyl, in healthy volunteers.^9–11^ These studies may provide a reference point for the research of fentanyl; however, the methods, including heat and cold pain threshold testing, used in these studies are usually complex and time-consuming, and thus not suitable for rapid preoperative pain measurement. In addition, other common effects of opioids including sedation were intentionally or unintentionally neglected in the previous studies. Therefore, it is necessary to establish a real-time approach to preoperatively determine the clinical effects of fentanyl.

Based on our knowledge and previous experience that the pressure pain threshold (PPT) test, a quantitative sensory type test, is rapid and reliable,^12–14^ we applied the PPT test to preoperatively detect the analgesic effects of fentanyl. Moreover, Narcotrend, an EEG index designed to measure the depth of anesthesia, has been confirmed to be effective as a continuous measure of the depth of sedation.^15–17^ By continuously monitoring the Narcotrend index (NTI), we were able to quantify the sedation effect of fentanyl. Therefore, a randomized, double-blinded, placebo-controlled study was conducted to explore the feasibility of detecting the effect of fentanyl and its individual differences in real time at 5 min and 10 min after drug administration using the PPT and NTI.

## MATERIALS AND METHODS

### Patients

The study was approved by the Hua Zhong University of Science and Technology Clinical Trial Ethnics Committee, and written informed consent was obtained from all patients prior to study enrollment. The study was registered at clinicaltrials.gov (ID: NCT11748071). In total, 80 patients (age range, 20–65 years) who had undergone elective surgery under general anesthesia were recruited for the present study. Because experimental pain measurement was found to be affected by sex,^18^ in this study only female patients were recruited to eliminate the effect of patient sex. This was a randomized, double-blinded, placebo-controlled trial. Before induction of anesthesia, in accordance with a random number table, the 80 patients were equally and randomly divided into 2 groups: the experimental (fentanyl [F]) group and the placebo (saline [S]) group. During the observation period, participants and observers were blinded to the random allocation sequence.

The inclusion criteria were as follows: (1) female patients; (2) right-hand-dominant; (3) non-smokers; and (4) body mass index of 20 to 30 kg/m^2^. The exclusion criteria were as follows: (1) known history of chronic pain; (2) use of any analgesic medication over the last 4 weeks; (3) alcohol or drug abuse; (4) presence of dermatitis or damaged, red, or swollen skin at the selected testing locations; (5) a known history of psychiatric diseases, communication disorders, diabetes mellitus, severe cardiovascular diseases, or kidney or liver diseases with compromised hepatic function; and (6) pre-oxygenation pulse oxygen saturation (SpO_2_) that could not be maintained at 90% or higher before induction of anesthesia.

### Study Design

Respiratory depression is known to be a serious and fatal adverse effect of opioids including fentanyl. In this study, all of the patients were observed before anesthesia induction in the operation room using well-equipped monitoring equipment, which not only ensured patient safety but also helped the investigators to obtain more comprehensive data on analgesic pharmacodynamics. Random allocation, participant inclusion, and intervention distribution were performed by different investigators.

**FIGURE 1:**
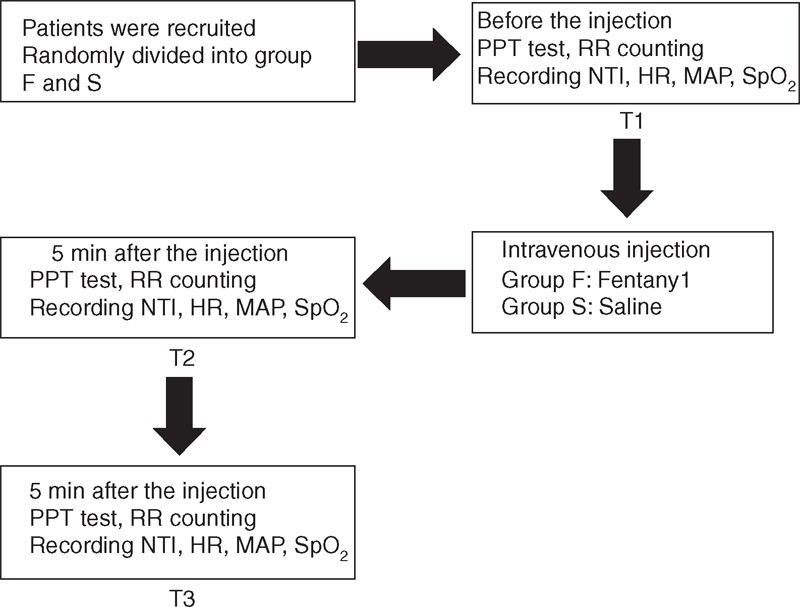
Flow diagram of the study procedure. HR = heart rate, MAP = mean arterial pressure, NTI = narcotrend index, PPT = pressure pain threshold, RR = respiratory rate.

The flow diagram of the study procedure is shown in Figure 1. After the patients entered the operating room, the electrocardiogram, blood pressure, and heart rate (HR) were monitored. The depth of sedation was monitored using Narcotrend (MonitorTechnik, Bad Bramstedt, Germany). All patients received pre-oxygenation treatment with 6 L/minutes oxygen using a face mask for 3 minutes before the study. Fentanyl or saline was injected intravenously according to the group allocation, with the patients and the investigators recording the data both blinded as to the group assignment. In Group F, 5 μg/kg fentanyl (the conventional dose) was diluted to 10 mL and then administered. In Group S, saline was administered in a volume of 10 mL to ensure injection of the same volume of liquid as in Group F. During the procedure, continuous oxygen was given to the patients to maintain SpO_2_, and the test was cancelled when SpO_2_ < 90%.

PPT was used to measure the analgesic effect, and the Narcotrend monitoring system was used to monitor the depth of sedation. Before (T1) and 5 minutes (T2) and 10 minutes (T3) after injection of fentanyl or saline, the PPT and respiratory rate (RR) of the patients were evaluated. The NTI, HR, SpO_2_, and blood pressure (analyzed as the mean arterial pressure [MAP]) were also recorded. At the end of the procedure, all of the patients were asked whether the study procedure caused any discomfort.

### Pressure Pain Threshold Measurement

**FIGURE 2:**
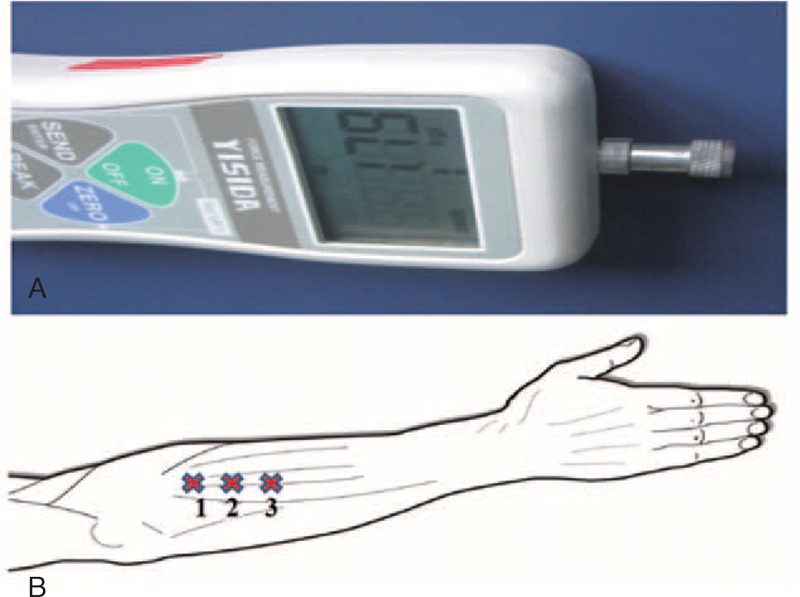
(A) YISIDA pressure algometer with a 1-cm^2^ probe (B) Testing locations 1, 2, and 3 (each marked with an X) on the lateral brachioradialis of the right elbow joint.

As in our previous studies,^19,20^ a hand-held electronic pressure algometer (YISIDADS2; Hong Kong, China; Figure 2A) was used to evaluate PPT. As shown in Figure 2B, 3 adjacent measurement points on the lateral brachioradialis of the right elbow joint that were separated from each other by 2 cm were selected as testing locations.^20,21^ The probe (1 cm^2^ size) was positioned perpendicularly to the skin surface, and the investigator applied continuous pressure at approximately the same rate (1 kg/s) according to the visual LCD display on the algometer. Furthermore, to prevent unnecessary tissue damage, the maximum force was limited to 15 kg, and the maximum force was recorded as a cut-off value.

Standardized instructions were given at the beginning of each testing session using the left forearm to familiarize the subject with the testing procedure. A standardized procedure was performed for all subjects; the subjects were asked to say “pain” when they started to feel pain (PPT) during the stimulation. The investigator applied the algometer to each of the 3 measurement points in the sequence shown in Figure 2B. The average value for the three measurements was calculated as usual. The subjects were asked to turn their heads to the opposite side of the testing limb so that they could not view the recorded values.

### Narcotrend Index Monitoring

The NTI determined by the Narcotrend monitoring system is a dimensionless continuous variable scored from 0 to 100 that reflects the depth of sedation.^16^ The Narcotrend system provides a six-stage classification from A (awake) to F (general anesthesia/coma). After injection of fentanyl or saline, we also recorded the initial time of NTI decline when the NTI changed from stage A (NTI ≥ 95) to B1 (NTI < 90) for every patient.

### Statistical Analysis

The required minimum sample size was 20 individuals per group, which was based on a randomized pilot study under a significance level of 0.01 and power of 0.9 using the sample size calculation software tool PASS version 11.0 (NCSS, Kayesville, UT).

All variables were summarized using standard descriptive statistics, such as the mean, standard deviation (SD), and frequency. An independent-sample *t* test was used to evaluate differences in age, weight, height, body mass index, PPT, NTI, RR, HR, MAP, and SpO_2_ at T1 between Groups F and S. Differences in the type of surgery between the 2 groups were analyzed using Pearson's chi-squared test. A two-way repeated-measures analysis of variance (ANOVA) followed by multiple comparisons (LSD testing) was used to evaluate the effects of time, group, and interaction.

In addition, absolute and percentage changes in the values of PPT and NTI at T2 and T3 compared to T1 in Group F were calculated. We then constructed a frequency histogram for the percentage changes in the 38 patients. A Pearson correlation analysis was applied to compare the absolute changes in the values of PPT with those of NTI in Group F. Statistical analyses were performed using SPSS for Windows version 17.0 (SPSS Inc., Chicago, IL), and two-tailed *P* < 0.05 was considered statistically significant.

## RESULTS

### General Results

Before the procedure, the NTI values in all patients were higher than 95. In Group F, 2 patients were excluded from the final analysis because SpO_2_ < 90%. The demographic data and preoperative observational index before the procedure in Groups F and S are shown in Table 1, and there was no significant difference between the 2 groups. In addition, none of the patients complained of any discomfort in the 10-minutes observation procedure.

**TABLE 1:**
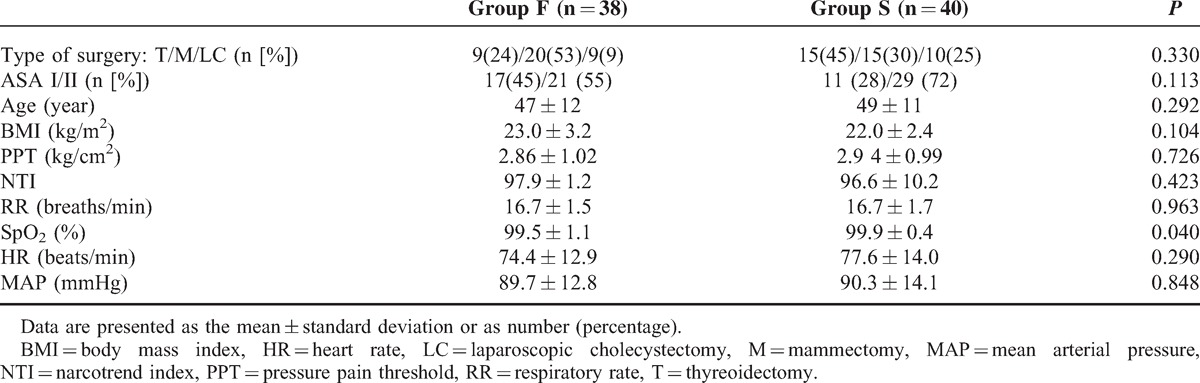
Patient Demographic Data and Preoperative Observational Index

### Measurement of Analgesic Effect

**FIGURE 3:**
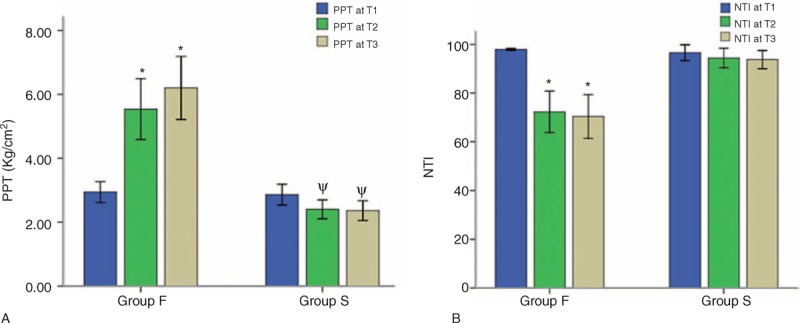
Comparison of PPT and NTI at different time points. NTI = narcotrend index; PPT = pressure pain threshold. ^∗^Significant difference (*P* < 0.001) between T3, T2, and T1; ψsignificant difference (*P* < 0.05) between T3, T2, and T1.

Two-way ANOVA for PPT showed that the group effect (*P* < 0.001), time effect (*P* < 0.001), and group and time interaction effect (*P* < 0.001) were statistically significant. As shown in Figure 3, in Group F, PPT at T2 (*P* < 0.001) and T3 (*P* < 0.001) was higher than that at T1, and no difference was observed for PPT between T3 and T2 (*P* = 0.247), whereas in Group S, PPT was lower at T2 (*P* = 0.037) and T3 (*P* = 0.023) than at T1. In addition, as shown in Table 2, PPT at T2 (*P* < 0.001) and T3 (*P* < 0.001) in Group F was higher than that in Group S.

**TABLE 2:**
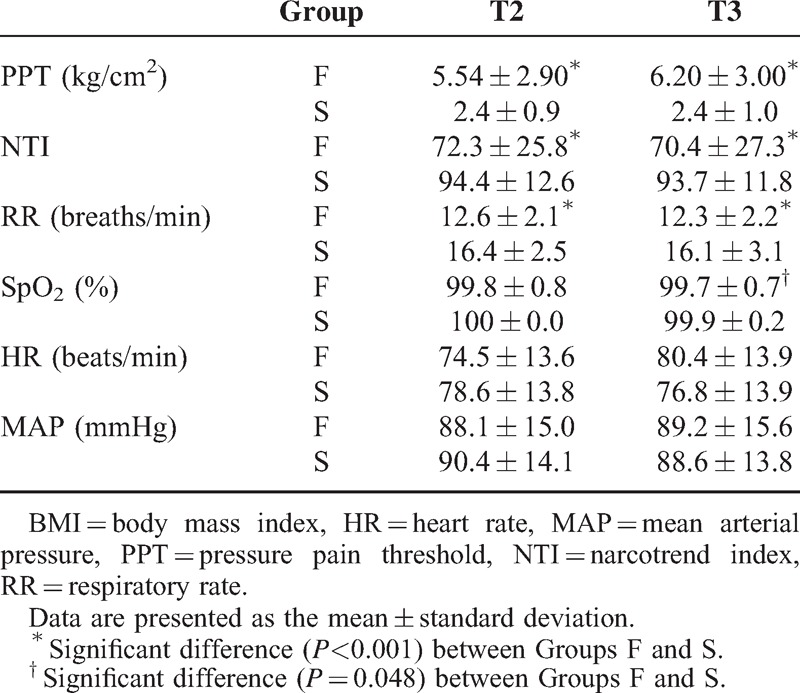
Comparison of Measurement Values After Fentanyl or Saline Injection Between Groups F and S

### Measurement of Sedative Effect

NTI in Group S did not vary over time (*P* > 0.05). However, in the two-way ANOVA for NTI of Group F, the group effect (*P* < 0.001), time effect (*P* < 0.001), and group and time interaction effect (*P* < 0.001) were statistically significant. In Group F, NTI was lower at T2 (*P* < 0.001) and T3 (*P* < 0.001) than at T1 but there was no significant difference (*P* = 0.700) between T2 and T3 (Figure 3). In addition, NTI in Group F at T2 (*P* < 0.001) and T3 (*P* < 0.001) was lower than that in Group S (Table 2). Furthermore, the initial time of the Narcotrend stage decrease from A to B in Group F patients was 314 ± 199 s with a variable coefficient of 63.4%, which may be considered moderate variation and represents the individual differences in the onset time of the sedative effect of fentanyl.

### Measurement of Respiratory and Other Parameters

In the two-way ANOVA for RR, the group effect (*P* < 0.001), time effect (*P* < 0.001), and group and time interaction effect (*P* < 0.001) were statistically significant. RR in Group F at T2 (*P* < 0.001) and T3 (*P* < 0.001) was lower than that in Group S. However, as shown in Table 2, in Group F, RR decreased significantly but remained normal (RR > 12) after fentanyl treatment. Two-way ANOVA for SpO_2_ indicated a statistically significant group effect (*P* = 0.004), whereas the time effect (*P* = 0.266) and group and time interaction effect (*P* = 0.492) were not significant. The SpO_2_ values were normal in both groups (Table 2). In addition, there were no significant differences in HR and MAP between Groups F and S.

### Changing Values of PPT and NTI

**FIGURE 4:**
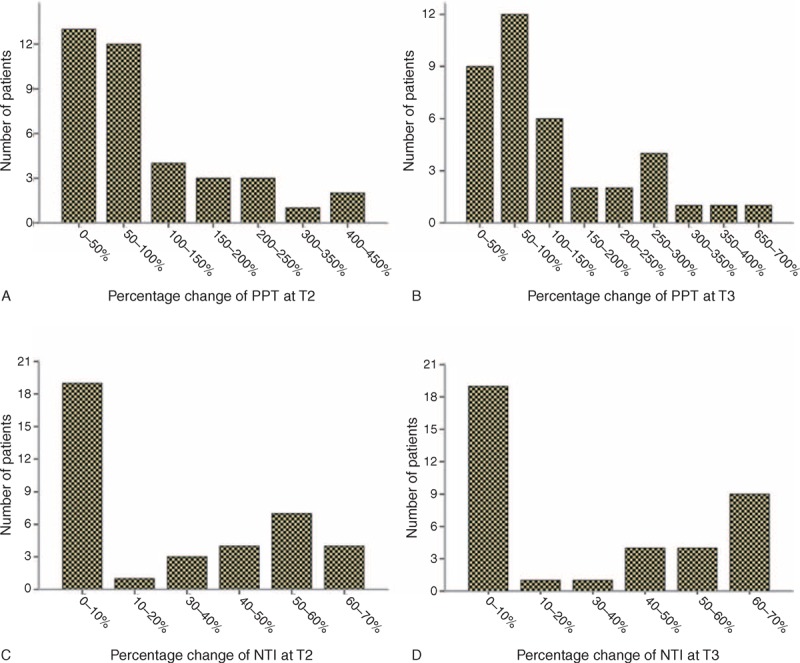
Distribution of the percentage changes of PPT and NTI. NTI = narcotrend index; PPT = pressure pain threshold.

We calculated the absolute and percentage changes in PPT and NTI at T2 and T3 relative to the baseline values at T1 in Group F. The absolute changes reflected the effect of fentanyl for every patient, whereas the percentage changes could also be used to compare the fentanyl sensitivity among different patients. A Pearson correlation analysis showed that the absolute changes in the values of PPT at T2 and T3 were significantly and positively correlated with changes in NTI (*r* = 0.444 at T2, *P* = 0.005; *r* = 0.332 at T3, *P* = 0.042). The distribution of the percentage change values is shown in Figure 4, which shows individual differences in the percentage changes of both PPT and NTI.

## DISCUSSION

This study demonstrated that a new real-time method could detect the analgesic and sedative effect of fentanyl using PPT and NTI prior to induction of anesthesia in the operation room.

To simplify the detection method, we made some modifications to the previously described PPT.^9,10,22–24^ First, the lateral brachioradialis of the right elbow joint was selected as the testing location because it was easy to expose and detect.^20^ Second, we applied a novel PPT testing procedure in which three adjacent measurement points at the testing location were measured separately, and then the average value of the three measurements of each measurement point was calculated. This was different from the conventional method in which the average value of the 3 measurements is calculated after 3 measurements at the same measurement point,^25^ as our method eliminates the requirement for time intervals between measurements.

In the current study, the PPT measurement required less than 1 minutes without causing any other discomfort for the patients, and thus the measurement was easily performed at the 3 observation time points with only a 5-minutes interval. The PPT values at 5 and 10 minutes after administration of fentanyl were significantly higher than those before administration, suggesting that the PPT value successfully reflected the analgesic effect of fentanyl. Furthermore, the distribution of the percentage changes of PPT values at T2 and T3 compared to T1 in patients of Group F represented individual differences in patients’ sensitivity to fentanyl. Therefore, the PPT testing applied in this study may be useful to rapidly detect analgesic effects and determine patients’ sensitivity to fentanyl.

Given the limitations of the Ramsay scale, eg, providing only an estimated range of the patient's sedation level^15^ and high subjectivity,^26^ we recorded NTI. In recent years, NTI has been shown to be an effective and accurate monitoring method for sedation evaluation.^15–17^ In our study, we also found that objective measurement of NTI from the patient's electroencephalographic data could be achieved without interviewer bias and that the NTI of patients in Group F significantly decreased with some individual differences at 5 and 10 minutes after administration of fentanyl. Thus, in the 10-minutes observation period, NTI successfully reflected the sedation effect of fentanyl.

In contrast to the PPT test, which has to be performed at a fixed observation time point, the use of Narcotrend enabled continuous monitoring of the sedation effect for patients. Absolute changes in the values of PPT and NTI were positively correlated, which suggests that the sedation effect detected by Narcotrend was consistent with the analgesic effect detected by PPT. Therefore, NTI may be useful to partly replace PPT for detection of the effect of fentanyl. Additionally, through continued monitoring by Narcotrend, we found that the initial time of the Narcotrend stage decrease in Group F patients was 314 ± 199 s with a variable coefficient of 63.4%, which represents individual differences in the onset time of the sedative effect and may also be used to measure the patients’ sensitivity to fentanyl. Based on the positive correlation between the PPT and NTI, we speculated that Narcotrend could potentially be applied to determine the patients’ sensitivity to fentanyl. Therefore, Narcotrend monitoring may be a good choice for evaluating the effect of opioids in the future.

Respiratory depression, which is one of the most serious adverse reactions for opioid analgesics,^27^ is defined as RR < 10 breaths/minute or SpO_2_ ≤ 90% for 3 minutes or more.^28^ Our results indicated a significantly decreased RR (even if at normal level) after administration of fentanyl. Therefore, monitoring of RR and SpO_2_ may be useful to assess ventilatory status in patients receiving fentanyl.

In this study, within a 10-minutes observation period for intravenous fentanyl before anesthesia induction, PPT and NTI significantly changed at T2 in Group F. The PPT value did not statistically increase from T2 to T3, and at the same time, no differences were found between T2 and T3 for NTI and RR. In addition, we found that the mean initial time of the Narcotrend stage decrease in Group F was 311 (95% CI: 248–374) seconds, indicating that 5 minutes after administration was suitable as the observation time point. Thus, a detection time of less than 10 minutes may be sufficient to test fentanyl efficacy using our method, allowing our methods to be convenient and rapid.

There were some limitations in the current study. First, because of the conventional induction dosage of fentanyl, respiratory depression was observed. As a matter of course, a reduced fentanyl dosage should be employed to ensure the safety of detecting method. Second, although our method had did not require time intervals between measurements, we found that the use of the 1-cm^2^ probe for pressure algometer measurement was inevitably time-consuming, as it consumed approximately 1 minutes for each testing procedure. Hence, the development of a more rapid method may be needed for more efficient measurement of pressure pain sensitivity.

In summary, in our study, PPT testing was used to successfully and rapidly detect the analgesic effect of fentanyl, and continuous NTI monitoring also detected the sedative effect in real time before induction of anesthesia in the operation room.

Additionally, a pressure algometer was easily and rapidly applied for evaluation of PPT, which is typically an acceptable method for patients, and Narcotrend provided continuous and objective data without disturbing the patients. Therefore, through the PPT and NTI, it was feasible to easily detect in real time the effect of fentanyl and its individual differences prior to induction of anesthesia in the operation room. Furthermore, this method could potentially be applied to preoperatively determine patients’ sensitivity to fentanyl and then facilitate the choice of the most effective postoperative personalized pain treatment plan. Additionally, this study may provide a new strategy for the evaluation of individualized perioperative medication with opioid analgesics.
